# Supplementary material for: Deletion of wfs1 Impairs Oligodendrocyte Precursor Cells Dorsal Distribution and Myelination Through the wfs1-hmgcs1 Axis in Zebrafish
Source: Biology (Basel). 2026 Aug 16;15(16):1402. doi: 10.3390/biology15161402 (PMC13510151; doi:10.3390/biology15161402)
Supplement: Supplementary file 1 [file biology-15-01402-s001.zip › File S1-Transcriptome sequencing data and differentially expressed genes (DEGs) in wfs1-- zebrafish at 5 dpf/2.KEGG/wfs1KOvsWT/GSEA/wfs1KO/STEROID_BIOSYNTHESIS/STEROID_BIOSYNTHESIS(DRE00100).html]

Details for gene set STEROID\_BIOSYNTHESIS(DRE00100)[GSEA]

|  || Dataset | deg.sample |
| Phenotype | sample.cls |
| Upregulated in class | wfs1KO |
| GeneSet | STEROID\_BIOSYNTHESIS(DRE00100) |
| Enrichment Score (ES) | 0.5722853 |
| Normalized Enrichment Score (NES) | 1.7428578 |
| Nominal p-value | 0.0 |
| FDR q-value | 0.10083144 |
| FWER p-Value | 0.17 |
Table: GSEA Results Summary

  

Fig 1: Enrichment plot: STEROID\_BIOSYNTHESIS(DRE00100)      
 Profile of the Running ES Score & Positions of GeneSet Members on the Rank Ordered List

  

| PROBE | DESCRIPTION (from dataset) | GENE SYMBOL | GENE\_TITLE | RANK IN GENE LIST | RANK METRIC SCORE | RUNNING ES | CORE ENRICHMENT || 1 | 565117 | cel.2 |  |  | 426 | 3.195 | 0.1374 | Yes |
| 2 | 322481 | cel.1 |  |  | 1043 | 2.053 | 0.2150 | Yes |
| 3 | 406662\_2 | msmo1 |  |  | 1111 | 1.984 | 0.3065 | Yes |
| 4 | 100004700 | cyp24a1 |  |  | 1170 | 1.931 | 0.3958 | Yes |
| 5 | 564424 | soat1 |  |  | 1916 | 1.404 | 0.4387 | Yes |
| 6 | 447891 | sc5d |  |  | 2509 | 1.170 | 0.4753 | Yes |
| 7 | 564868 | soat2 |  |  | 2590 | 1.147 | 0.5269 | Yes |
| 8 | 494054 | tm7sf2 |  |  | 3158 | 1.001 | 0.5564 | Yes |
| 9 | 406662 | msmo1 |  |  | 3921 | 0.844 | 0.5723 | Yes |
| 10 | 494102 | dhcr24 |  |  | 5884 | 0.598 | 0.5390 | No |
| 11 | 368360 | lbr |  |  | 10303 | 0.285 | 0.4137 | No |
| 12 | 406713 | lipf |  |  | 11003 | 0.244 | 0.4033 | No |
| 13 | 436600 | ebp |  |  | 11261 | 0.230 | 0.4061 | No |
| 14 | 793309 | cyp27b1 |  |  | 11749 | 0.203 | 0.4004 | No |
| 15 | 414331 | cyp51 |  |  | 12077 | 0.186 | 0.3989 | No |
| 16 | 563369 | cyp2r1 |  |  | 16792 | -0.026 | 0.2523 | No |
| 17 | 768185 | hsd17b7 |  |  | 20645 | -0.196 | 0.1406 | No |
| 18 | 571911 | fdft1 |  |  | 22728 | -0.309 | 0.0899 | No |
| 19 | 378446 | dhcr7 |  |  | 22772 | -0.312 | 0.1033 | No |
| 20 | 565329 | sqleb |  |  | 25077 | -0.452 | 0.0523 | No |
| 21 | 795400 | lss |  |  | 25608 | -0.494 | 0.0590 | No |
| 22 | 550369 | nsdhl |  |  | 28663 | -0.849 | 0.0032 | No |
| 23 | 799528 | sqlea |  |  | 29200 | -0.974 | 0.0324 | No |
| 24 | 494054\_1 | tm7sf2 |  |  | 29622 | -1.102 | 0.0712 | No |
Table: GSEA details [plain text format]

  

Fig 2: STEROID\_BIOSYNTHESIS(DRE00100)      
 Blue-Pink O' Gram in the Space of the Analyzed GeneSet

  

Fig 3: STEROID\_BIOSYNTHESIS(DRE00100): Random ES distribution      
 Gene set null distribution of ES for **STEROID\_BIOSYNTHESIS(DRE00100)**
